# A Low-Rank Matrix Recovery Approach for Energy Efficient EEG Acquisition for a Wireless Body Area Network

**DOI:** 10.3390/s140915729

**Published:** 2014-08-25

**Authors:** Angshul Majumdar, Anupriya Gogna, Rabab Ward

**Affiliations:** 1 Indraprastha Institute of Information Technology, Delhi 110020, India; E-Mail: anupriya@iiitd.ac.in; 2 Department of Electrical and Computer Engineering, University of British Columbia; Vancouver V6T1Z4, Canada; E-Mail: rababw@ece.ubc.ca

**Keywords:** EEG, WBAN, compressed sensing, low-rank matrix recovery

## Abstract

We address the problem of acquiring and transmitting EEG signals in Wireless Body Area Networks (WBAN) in an energy efficient fashion. In WBANs, the energy is consumed by three operations: sensing (sampling), processing and transmission. Previous studies only addressed the problem of reducing the transmission energy. For the first time, in this work, we propose a technique to reduce sensing and processing energy as well: this is achieved by randomly under-sampling the EEG signal. We depart from previous Compressed Sensing based approaches and formulate signal recovery (from under-sampled measurements) as a matrix completion problem. A new algorithm to solve the matrix completion problem is derived here. We test our proposed method and find that the reconstruction accuracy of our method is significantly better than state-of-the-art techniques; and we achieve this while saving sensing, processing and transmission energy. Simple power analysis shows that our proposed methodology consumes considerably less power compared to previous CS based techniques.

## 1. Introduction

This work addresses the problem of energy efficient sensing, processing and transmission of EEG signals in a wireless body area network (WBAN) context. In these applications, the battery energy is limited. The problem is to minimize the energy consumed in order for the battery to last longer. The energy is consumed by sensing (sampling) the physiological wave, processing the sensed data, and wirelessly transmitting them to a nearby base station. At the base station the original signal is restored and the information required is extracted from the signal depending on the application (seizure detection, sleep apnea study, brain computer interface, mood detection, *etc.*).

Recently, compressed sensing (CS) based techniques have been gaining popularity in addressing this problem [1–6]. However, CS based techniques can only reduce the transmission and the processing (*i.e.* data compressing) energy cost. Unfortunately, existing CS techniques cannot reduce the energy cost for sensing the EEG signal.

To reduce the transmission cost, one should transmit as little bits as possible, *i.e.*, the signal should be compressed prior to its transmission. The use of compressed sensing is ideal for compressing a signal when the computational power is at a premium. Traditionally, compression techniques have assumed that the encoder is computationally powerful whereas the computational power at the decoder is limited. Such a scheme is a perfect fit for multimedia transmission, where the encoder is powerful but the decoder (TV, phones, *etc.*) is not so computationally endowed. The problem in WBAN is exactly the opposite. The computational power at the sensor nodes is limited, but the processors at the receiving base station are powerful. Thus, CS techniques are best for WBANs applications since its encoding-decoding scheme requires little power at the transmitter but needs much computational effort at the receiver/decoder.

At present, the EEG signal is first fully sampled. To obtain the CS compressed samples, the fully sampled signal is projected linearly onto a lower dimension, thereby compressing the signal. Finally, the compressed signal is transmitted. Since the projection is linear, the computational/processing cost is low—can be performed on simple DSP chips. CS requires a smart decoder and needs solving an optimization problem in order to recover the EEG signal from the lower dimensional projections.

As mentioned before, CS does not reduce the energy consumed by sensing and processing. This study addresses the problem of reducing the sensing energy. For reducing sensing energy, we need to under-sample the signal. In such a scenario it is not judicial to employ CS based techniques owing to certain theoretical limitations that will be discussed later in the paper. As we propose to reduce the sensing energy by just (randomly) under-sampling the EEG signal, we do not fully sample the signal, the sensing energy is naturally reduced. Therefore we will also not require any further energy to compress the data. The signal is automatically compressed as it is sensed.

To recover the EEG signals, one may use compressive sampling techniques. However, these will not yield the best results. This is explained in detail in Sections 2 and 3 below. In brief, CS has two requirements—sparsity of the signal and incoherence between the sampling basis and the transform used to sparsify the signal. In this paper, the proposed sampling basis is the Identity/Dirac basis. Generally wavelet, DCT or Gabor dictionary is used as the sparsity basis in CS for EEG signals. Unfortunately none of these are incoherent with the Dirac basis and are thus not expected to give the best results. The other option is to use Fourier as the sparsity basis, because Fourier is maximally incoherent with the Dirac basis. However, the Fourier transform (coefficients) of EEG signals are not very sparse. Therefore, the Fourier sparsifying basis will not give good recovery results also. In short, there is no sparsifying basis that satisfies both the requirements of sparsity and incoherence.

To reconstruct the original EEG signals from the (randomly) acquired samples, we note that the EEG signals from the various channels/probes are correlated with each other. Thus, the matrix formed by the ensemble of signals from all the channels have dependent columns, thereby it constitutes a low-rank matrix. As the EEG signals are only partially sampled at the sensing node, the base station receives a partially observed signal ensemble matrix. To reconstruct the low-rank signal ensemble matrix, we invoke matrix completion techniques. This is the first time, a low-rank matrix recovery technique is used for recovering EEG signals.

The contributions of prior works are discussed in Section 3. Since these techniques are all based on CS, we first give a brief introduction to CS. This introduction will help us understand the shortcomings of this approach in solving our problem. The proposed formulation is described in Section 3. The algorithm for matrix completion is derived in Section 4. We discuss the experimental results in Section 5. Finally the conclusions of this work are discussed in Section 6.

## 2. Brief Review of Compressed Sensing

Compressed Sensing (CS) studies the problem of solving an under-determined system of linear equations when the solution is known to be sparse:

(1) $$\begin{array}{cc} y_{m\times 1}=A_{m\times n}x_{n\times 1}, & m<n \end{array}$$

The solution is assumed to be k-sparse, *i.e.*, it has only k non-zero values.

Theoretical studies in CS [1,2] have shown that, it is possible to recover the sparse solution to the under-determined system of equations by solving the following *l_1_*-minimization problem:

(2) $$\underset{x}{\min}{\left\|x\right\|}_{1}\;\text{subject to}\;y=Ax$$

In most cases, one has to deal with a noisy system of equations, *i.e.*,

(3) $$y=Ax+\eta ,\eta ∼N(0,\sigma^{2})$$

In such a case, the equality constraint is relaxed and in order to recover *x* one solves the following instead;

(4) $$\underset{x}{\min}{\left\|x\right\|}_{1}\;\text{subject to}\;{\left\|y-Ax\right\|}_{2}^{2}\leq \varepsilon ,\varepsilon =m\sigma^{2}$$

For practical problems, the signal of interest is never sparse (except in few isolated cases). However a large number of natural signals are sparse in a transform domain, e.g., speech is sparse in short time Fourier transform, EEG is sparse in Gabor, MR images are sparse in wavelets *etc.* When the sparsifying transform is orthogonal or tight-frame, it is possible to express the signal via the analysis and the synthesis equations:

(5) $$\begin{array}{ll} \text{analysis}: & \begin{array}{cc} \alpha & =\Psi x \end{array}\\ \text{synthesis}: & \begin{array}{cc} x & =\Psi^{T}\alpha \end{array} \end{array}$$

where *x* is the signal (not sparse), Ψ is the sparsifying transform (orthogonal or tight-frame) and α is the sparse transform coefficient vector.

In such a scenario, it is possible to express (3) in the following form by incorporating the sparsity promoting transform (synthesis):

(6) $$y=A\Psi^{T}\alpha +\eta$$

Since, α is sparse, the signal can be recovered by *l_1_*-minimization;

$$\underset{\alpha }{\min}{\left\|\alpha \right\|}_{1}\;\text{subject to}\;{\left\|y-A\Psi^{T}\alpha \right\|}_{2}^{2}\leq \varepsilon$$

In CS terminology, A is referred to as the measurement operator, y is the measurement and Ψ is the sparsifying basis/transform.

The form (7) is known as the synthesis prior formulation. When the sparsifying transform is not orthogonal or tight-frame, e.g., Gabor, the analysis equation remains the same but the synthesis form does not hold.

$$\begin{array}{ll} \text{analysis}: & \begin{array}{cc} \alpha & =\Psi x \end{array}\\ \text{synthesis}: & \begin{array}{cc} x & \neq \Psi^{T}\alpha \end{array} \end{array}$$

In such a situation, it is not possible to frame a synthesis prior formulation as before; one needs to formulate the recovery as an analysis prior problem [3,4]:

(7) $$\underset{x}{\min}{\left\|\Psi x\right\|}_{1}\;\text{subject to}\;{\left\|y-Ax\right\|}_{2}^{2}\leq \varepsilon$$

The analysis prior is a more general formulation. It can be applied to both orthogonal/tight-frame as well as other transforms. In the special case that the transforms are orthogonal or tight-frame the analysis and the synthesis prior theoretically yields the same results.

Before concluding our discussion on CS, we briefly discuss the relationship between the measurement operator and the sparsifying transform. In the ideal case, where the original signal itself is sparse, the number of measurements required to recover the solution is given by:

(8) m=Cklogn

However, when the solution is not sparse in the signal domain, but sparse in a transform domain, the number of measurements required is given by [5]:

(9) m=Cμklogn

where μ is the coherence between the measurement operator and the sparsifying basis.

When the measurement operator A is the Canonical/Dirac basis and the sparsifying basis is the Fourier, the minimum value of μ (unity) is achieved; for any other pair of basis, μ is higher. This implies that, the greater the coherence between the two bases, the higher is the number of measurements required for recovery. In practical situations, the number of measurements is limited, thus, the greater the coherence, the worse is the recovery and vice versa.

The above gives us a practical guideline on how to select the sparsifying basis. Generally, the measurement operator is fixed for a given problem (dictated by the physics), but we have flexibility in choosing the sparsifying basis. We would like to choose a basis that gives a very sparse representation of the signal; since a sparser signal means better recovery (if the number of measurements are fixed). However, we should also be careful about the incoherence of the sparsifying basis and the measurement operator; it should be kept low, otherwise the recovery results will deteriorate.

## 3. Literature Review

One of the earliest works that applied CS for EEG signal compression and transmission is [6]. The EEG signal is projected onto an i.i.d Gaussian basis for compression:

(10) b=Mx+η

where *x* is the fully sampled EEG signal, *M* is the measurement basis (Gaussian) and *b* is the compressed signal.

In [6] it is assumed that the EEG signal is sparse in Gabor basis. However, they posed a synthesis prior problem using the Gabor basis, *i.e.*,

(11) $$\underset{\alpha }{\min}{\left\|\alpha \right\|}_{1}\;\text{subject to}\;{\left\|b-M\Psi^{T}\alpha \right\|}_{2}^{2}\leq \varepsilon$$

As discussed in the previous section, this formulation will not yield the best results theoretically, since the analysis-synthesis pair (5) do not hold for Gabor transform.

In [7], different sparsifying transforms were compared—wavelets, Gabor, spline- for recovering the EEG signal using the synthesis prior problem. They found that the Gabor basis yields the best recovery results.

The possibility of exploiting inter-channel correlation in order to improve EEG signal reconstruction was mentioned in [6]. This problem was then partially addressed in [8]. In [8], the inter-channel correlation was not modeled, instead a joint reconstruction problem was framed where the signals from all the channels were reconstructed simultaneously. This work used wavelets as the sparsifying basis. The other departure from prior studies is in terms of the measurement operator. Previous studies [6,7] used i.i.d Gaussian matrices; these are theoretically good, but not very practical because these matrices are dense, so storing them and operating with them is not efficient. In [8], a random binary ensemble (consisting of few one's and rest zeroes) was used instead—such a measurement operator is computationally and operationally efficient.

Let us analyze the methodology in [8] more analytically. The measurement operation in [8] remains the same:

(12) $$b_{c}=M_{c}x_{c}+\eta ,\forall c$$

Here c denotes the c^th^ channel (and there are C channels). The measurement operator (compressing basis) M_c_ is a binary matrix. It is assumed in [8] that the EEG signal is sparse in wavelet basis; this allows for incorporating the synthesis Equation (5) into (12) resulting in:

(13) $$b_{c}=M_{c}\Psi^{T}\alpha_{c}+\eta ,\forall c$$

This can be represented more compactly as follows:

(14) vec(B)=Φvec(Z)+vec(N)

Here B and Z are formed by stacking the b_c_'s and the α_c_'s as columns, Φ is a block diagonal matrix formed by M_c_Ψ^T^ along the blocks. In [8], it is argued that since each of the α_c_'s are sparse, Z is sparse as well and hence can be recovered by *l_1_*-minimization:

(15) $$\underset{Z}{\min}{\left\|\text{vec}(Z)\right\|}_{1}\;\text{subject to}\;{\left\|\text{vec}(B)-\Phi \text{vec}(Z)\right\|}_{2}^{2}\leq \varepsilon$$

Since the wavelet transform is orthogonal, the EEG signals can be recovered from Z by applying the synthesis equation. Although this work jointly solves for all the EEG signals from all the channels; it does not exploit the channel correlations explicitly.

A recently proposed EEG signal model assumes a block structure of the EEG signals in a transform domain (DCT or wavelet) [9]. Although there is no theoretical or physical intuition behind this assumption; it is shown in [9] that a Block Sparse Bayesian Learning (BSBL) algorithm yields good recovery results.

An efficient CS framework for EEG signal transmission over WBAN is proposed in [10]. This study proposes an end-to-end solution. It samples the full signal, but instead of compressing the full signal it subtracts the mean from each signal. It then exploits both the temporal correlation within EEG signals and the spatial correlations amongst the EEG channels. By combining transform coding techniques with CS, this work was able to achieve up to 8-fold compression.

There is an interesting approach to the EEG monitoring problem proposed in [11]. Here, the EEG signals are sampled fully and then compressed on a suitable basis. However, instead of sensing the compressed signal, the authors show that some basis analysis (like seizure detection) can be performed in the compressed domain at the nodes itself. This precludes the necessity to transmit the signal which in turn reduces communication cost. This is a smart approach but is limited in its application. First, even for EEG patient monitoring applications, the idea of detecting seizures by a low-power computer is unacceptable. One would like a medical practitioner to take such decisions—this would require the signal to be transmitted. Second, for other applications like BCI, the EEG signal needs to be transmitted, there is no other option—a proposal like [11] will not work in such a scenario.

All the previous techniques operated on a single channel of EEG. They did not account for inter-channel correlations. In a recent study [12], we showed that it is possible to improve the reconstruction results even further by accounting for inter-channel correlations. As the channels are correlated, the sparsity pattern is repeated across all channels. This observation led to the formulation of a row-sparse MMV recovery problem for recovering the full multi-channel EEG signal ensemble.

These prior studies do not follow the true philosophy of CS; the idea behind CS is to reduce the sampling requirements (compared to the Nyquist rate). These studies in EEG [6–12] do not follow this basic tenet of CS. All of them sample the full signal (at Nyquist rate) and compress them. In our proposed approach, we follow the CS philosophy—we under-sample the EEG signal directly thereby reducing the sampling requirements. However, our signal recovery process does not follow sparsity based CS techniques. We used matrix completion based approaches. The detailed approach is discussed in the following section.

## 4. Proposed Approach

All prior studies only reduced the processing and transmission costs; they sampled the full signal (therefore did not reduce the sensing and processing energy) and compressed it by projecting onto a lower dimension using Gaussian/Binary matrices. In this work, our aim is to reduce the sensing and processing energy as well. This is achieved by acquiring less samples for each EEG channel. The samples are acquired at random time instants. The data acquisition for the c^th^ channel can be modeled as:

(16) $$b_{c}=R_{c}x_{c}+\eta ,\forall c$$

where R is a uniform random sampling mask.

In this scenario, the acquired signal (b) is inherently compressed (compared to *x*). Thus, we do not need to expend any energy in processing *i.e.*, compressing the signal. This is the additional benefit of our proposed acquisition scheme.

**Figure 1.**
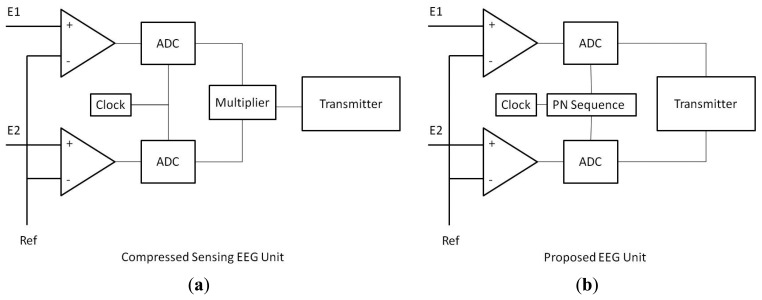
Compressed Sensing *vs.* Proposed EEG Acquisition and Transmission.

The schematic design of the CS *vs.* proposed units are shown in Figure 1. In the CS unit, the full signal is sampled. After that the signal is compressed by projecting onto a lower dimension; this requires a DSP chip for multiplication which consumes considerable amount of power. The compressed EEG signal is finally transmitted.

The clock dictates the sampling rate of the ADC. Our proposed modification requires a pseudo-random sequence (pn sequence) generator between the clock and the ADC (Analog to Digital Converter). The pn sequence determines if the clock will sample at a particular instant or not. Effectively this scheme under-samples the signal randomly. Moreover, since signal is inherently under-sampled there is no need to compress it. This significantly reduces the processing cost.

The main question is: **How do we reconstruct the under-sampled EEG signal?** Since Compressed Sensing based techniques have been successfully applied in the past for recovering EEG signals compressed by binary/Gaussian basis, the natural response would be to use CS to recover the signal. However a deeper analysis reveals that this will not be possible. The answer lies in the concluding remarks of Section 2. To recover the signal we need to apply a sparsifying transform that yields a sparse representation of the signal; but at the same time the chosen sparsifying basis should be maximally incoherent with the measurement operator (in this case a Dirac basis). We mentioned that the Dirac basis is perfectly incoherent with the Fourier transform. However, unfortunately the Fourier transform does not lead to a very sparse representation of the EEG signal: hence it is not an acceptable choice. On the other hand, the wavelet and Gabor basis yield a sparse representation of EEG signals, but are not incoherent with the Dirac basis (measurement operator)—hence they cannot be employed either as a sparsifying transform. Therefore, in short, CS does not seem to be a good option for recovering EEG signals that are acquired via random under-sampling as proposed in this paper. We carried out experiments to see whether well known CS techniques can be used in practice to recover EEG signals from their under-sampling measurements; we found that the recovery from such methods is very poor and hence do not report the results.

We propose an alternate approach. The multi-channel data acquisition model (16) can be succinctly represented as follows:

(17) B=R⊙X+N

where B and *X* are formed by stacking the *b_c_*'s and *x_c_*'s as columns; *R* is the binary sampling mask for all the EEG signals composed of *R_c_*'s along the columns and ⊙ denotes binary multiplication.

**Figure 2.**
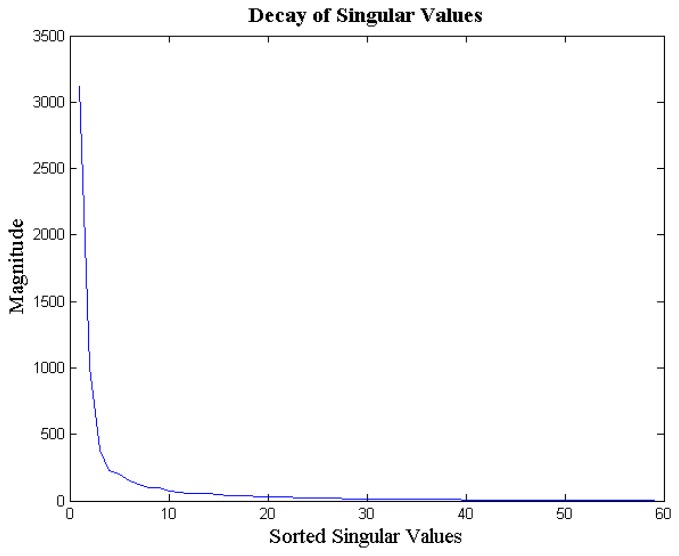
Decay of singular values for a multi-channel signal ensemble.

The EEG signals from different channels are correlated with each other; this is because all the EEG probes sense the same brain activity. Since the signal acquired by the different channels are correlated, the columns of the matrix *X* are not linearly independent from each other. Thus, the multi-channel EEG signal ensemble *X* is a rank-deficient matrix. We empirically verified our claim. For each signal ensemble of the BCI competition database, we computed the ratio of energy concentrated in the top 10 singular values to the total energy of the ensemble. We found that, on an average, about 97% of the EEG signal ensemble's energy is concentrated in the top 10 singular values; the standard deviation is 2%. In Figure 2 we show the decay of the singular values of a typical EEG signal ensemble (from BCI Competition database). As can be seen, the decay is very sharp; implying that the matrix *X* (EEG signal ensemble) is indeed approximately rank deficient.

We have showed that our problem satisfies the first criteria for matrix completion—our EEG signal ensemble matrix is low-rank. However, to be a candidate for matrix completion, we also need to check for the incoherence of the singular vectors. In a recent theoretical work [13] it says that when the matrix satisfies the following standard incoherence condition with parameter μ_0_,

$$\begin{array}{ll} \underset{i}{\max}{\left\|U^{T}e_{i}\right\|}_{2} & \leq \sqrt{\frac{\mu_{0}r}{n_{1}}}\\ \underset{j}{\max}{\left\|V^{T}e_{j}\right\|}_{2} & \leq \sqrt{\frac{\mu_{0}r}{n_{2}}} \end{array}$$

It is possible to recover the matrix with $m\geq c\frac{\mu_{0}r\log^{2}(n_{1}+n_{2})}{\min\{n_{1},n_{2}\}}$ measurements. Here *e*'s represent the canonical basis.

It is easy to verify that the value of the parameter μ_0_ varies from 1 to max(n_1_,n_2_)/r. We tested on the BCI competition dataset and found that the average value of μ_0_ is 3.32 which is much smaller than the minimum upper bound of 6.4 (64 channels and assuming a rank of 10). The maximum upper bound is 300 (3000 time points and rank 10).

We have thus verified that recovering *X* from (17) is a classic low-rank matrix completion problem. Such a problem arises in several branches of computer science and signal processing, e.g., in collaborative filtering and sensor localization. In all these problems (including ours), one ideally needs to solve for a low-rank matrix subject to data constraints:

(18) $$\underset{X}{\min}\text{rank}(X)\;\text{subject to}\;{\left\|B-R⊙X\right\|}_{\text{Fro}}^{2}\leq \varepsilon$$

where “*Fro*” denotes the Frobenius' norm.

Unfortunately, this is an NP hard problem. Theoretical studies in low-rank matrix completion [12] prove that it is possible to recover the correct solution (low-rank matrix) by relaxing the NP hard norm minimization problem by its nearest convex surrogate the Nuclear Norm (sum of singular values):

(19) $$\underset{X}{\min}{\left\|X\right\|}_{*}\;\text{subject to}\;{\left\|B-R⊙X\right\|}_{\text{Fro}}^{2}\leq \varepsilon$$

This is a convex problem and can be solved via Semi-Definite Programming (SDP).

In CS [14,15], it was shown that better recovery can be obtained when non-convex *l_p_*-minimization (0 < p < 1) is used instead of the convex *l_1_*-minimization (4). Although there are no theoretical results in non-convex Schatten-p norm minimization for low-rank matrix completion, an empirical study in [16] have shown that the recovery results are indeed an order of magnitude better compared to state-of-the-art nuclear norm minimization techniques. Therefore, we here propose solving for the EEG matrix ensemble via the Schatten-p norm optimization problem,

(20) $$\underset{X}{\min}{\left\|X\right\|}_{S_{p}}^{p}\;\text{subject to}\;{\left\|B-R⊙X\right\|}_{\text{Fro}}^{2}\leq \varepsilon$$

where the Schatten-p norm is defined as the *l_p_*-norm of the singular values.

In our prior work [16], we have developed an algorithm for solving matrix completion algorithms. It gives good results for small scale problems but is not scalable. In this paper, we derive a new scalable algorithm to solve (20) in the following section. However, before getting into the derivation let us analyze the power savings our method offers compared to previous CS based techniques.

### Power Analysis

We have argued in principle how our proposed technique will reduce the energy consumed by sensing the signal and eliminate that needed for processing (compression). In this sub-section we will analyze the overall energy saving by following a power model proposed in [17]. There are some other works like [18] which have done power analysis; but such studies are not as thorough (in terms of power analysis) as [17].

First we will discuss the power consumption for the CS EEG unit. The total power as discussed before is comprised of three major factors:

(21) $$P_{\text{tot}}=P_{\text{sense}}+P_{\text{proc}}+P_{\text{comm}}$$

The sensing is comprised of two portions—amplification (*P_amp_*) and analog-to-digital conversion (*P_ADC_*) (see Figure 1); therefore *P_sense_* = *C*(*P_amp_* + *P_ADC_*), where C is the total number of channels.

The processing consists of two operations—random number generation (*P_RNG_*) and matrix-vector multiplication (*P_mult_*); thus, *P_proc_* = *P_RNG_* + *P_mult_*. It is difficult to uniquely model the power requirement for communication (transmission) because it is dependent on the communication protocol. In general the communication power is expressed as *P_comm_* = *CJf_s_R* where *J* is the transmission power per bit, *f_s_* is the sampling frequency (bits per second) of the ADC and *R* is the number of bits per sample (resolution).

The sensing power and the communication power increases linearly with the number of channels, but the processing power does not scale with the number of channels. CS based techniques can only reduce the transmission power by compressing an *n* dimensional signal to *m* samples via matrix vector multiplications. Thus, the total power consumption by a CS based EEG WBAN will consume:

(22) $$P_{\text{tot}}=C(P_{\text{amp}}+P_{\text{ADC}})+P_{\text{RNG}}+P_{\text{mult}}+C\frac{m}{n}Jf_{s}R$$

Following the same arguments, our proposed unit will only consume (see Figure 1):

(23) $$P_{\text{tot}}=C(P_{\text{amp}}+\frac{m}{n}P_{\text{ADC}})+C\frac{m}{n}Jf_{s}R$$

The amplifier being an analog device cannot be switched on and off. We do not need the matrix-vector product for compression so the corresponding power terms vanish; we compress right when we sample. The transmission power remains the same as that of a CS based unit.

Establishing hard values for power consumption gives some idea regarding the actual power cost and how much we save. For the ADC, we get the values from [19]–for a 12 bits per sample (*R*), 0.5 kSample/sec sampling rate (*f_s_*) and P_ADC_ = 0.2 μW. The specifications of the power amplifier (for EEG signals) is obtained from [20,21]—30 dB gain and 30 Hz bandwidth require P_amp_ = 0.9 μW. The power consumption for the random number generator is 3 μW [20]. In [16] the power cost of matrix vector multiplication is pegged at 352 μW assuming that the DSP chip is of TI MSP430 family. The transmission energy required per bit is estimated to be 5nJ per bit [17].

With these figures, we compute the energy requirement for a CS based system using (22). 64×(0.2+0.9)+3+352+64×0.2×0.5×5×12 ≡ 809 *μW*. Here it is assumed that the compression ratio is 5:1 (0.2).

Now, considering our proposed methodology, the power consumption (for same compression ratio) is computed using (23): 64×(0.2*0.2+0.9)+64×0.2×0.5×5×12 ≡ 444 *μW*; this is about half the power consumption required by CS techniques. In other words, with our proposed methodology, the WBAN is expected to last almost twice longer. If the sampling ratio is increased to 5:2 (0.4), the power requirement for the CS based method is 1.19 mW and that of our proposed technique is 0.83 mW.

It should be noted that the analysis has been carried out using the model proposed in [17]. The values that correspond to the different units are from different papers; they are not on the same technology. Hence, these values are suggestive and should not be compared with the real system.

## 5. Optimization Algorithm

The task is to solve the optimization problem (20):

$$\underset{X}{\min}{\left\|X\right\|}_{S_{p}}^{p}\;\text{subject to}\;{\left\|B-R⊙X\right\|}_{\text{Fro}}^{2}\leq \varepsilon$$

Solving the constrained problem directly is hard. Therefore, we propose to solve its unconstrained counterpart instead.

(24) $$\underset{X}{\min}{\left\|B-R⊙X\right\|}_{\text{Fro}}^{2}+\lambda {\left\|X\right\|}_{S_{p}}^{p}$$

The constrained (20) and the unconstrained (24) versions are equivalent for the correct choice of the parameters *λ* and *ε*. Unfortunately, the relationship between *λ* and *ε* is not analytical Therefore, in this work, we propose to solve the constrained problem by iteratively solving the unconstrained version with progressively smaller values of *λ*. Such a type of cooling technique has been successfully used before for solving constrained problems [16].

We propose to solve (24) using a Bregman type variable splitting algorithm with Alternating Directions Multiplier Method (ADMM) [22]. We introduce a proxy variable *P* for *X*. We add a term relaxing the equality constraints of each quantity and its auxiliary variable, and in order to enforce equality at convergence, we introduce Bregman variable *Bp*. Thus, the problem (24) is recast as:

(25) $$\underset{X,P}{\min}{\left\|B-R⊙X\right\|}_{\text{Fro}}^{2}+\lambda {\left\|P\right\|}_{S_{p}}^{p}+\gamma {\left\|P-X-Bp\right\|}_{\text{Fro}}^{2}$$

This allows the problem to be split into an alternating minimization of the following sub-problems:

(26) $$\underset{X}{\min}{\left\|B-R⊙X\right\|}_{\text{Fro}}^{2}+\gamma {\left\|P-X-Bp\right\|}_{\text{Fro}}^{2}$$

(27) $$\underset{P}{\min}\lambda {\left\|P\right\|}_{S_{p}}^{p}+\gamma {\left\|P-X-Bp\right\|}_{\text{Fro}}^{2}$$

It is easy to solve (26) since it is an *l_2_*-minimization problem. Conjugate Gradient (CG) is used to solve them efficiently.

The sub-problem (27) is a standard Schatten-p norm minimization and can be solved efficiently using p-shrinkage of singular values [16,19]:

(1). Compute SVD of *X+Bp*, *i.e.*, *U*Σ*V^T^= X+Bp*.
(2). Soft threshold the singular values: $\hat{\sum }=\text{signum}\left(\text{diag}(\sum )\right)\left(0,\text{diag}(\sum )-\frac{\lambda }{2\alpha }\text{diag}(\sum )\cdot^{\left(p-2\right)}\right)$.
(3). Low-rank approximation: *P* = *U*Σ̂*V^T^*.

Here *diag*(Σ)·^(*p*−2)^ means that each of the diagonal elements in Σ (singular values) is raised to the said power (*p*−*2*).

In the final step, the Bregman relaxation variables need to be updated:

Bp=Bp+X−P

Putting everything together, the complete algorithm to solve the unconstrained problem (22) is given as follows:

At each iteration (k):
$X_{k}\leftarrow \underset{X}{\min}{\left\|Y-M(X)\right\|}_{\text{Fro}}^{2}+\gamma {\left\|P-X-Bp\right\|}_{\text{Fro}}^{2}$
$P_{k}\leftarrow \underset{P}{\min}\lambda {\left\|P\right\|}_{S_{p}}^{p}+\gamma {\left\|P-X-Bp\right\|}_{\text{Fro}}^{2}$  Compute SVD of *X* + *Bp, i.e., U*Σ*V ^T^* = *X_k_*_−1_ + *Bp_k_*_−1_.  Soft threshold the singular values: 
$\hat{\sum }=\text{sigum}(\text{diag}(\sum ))(0,\text{diag}(\sum )-\frac{\lambda }{2\alpha }\text{diag}(\sum ).^{\left(p-2\right)})$ Low-rank approximation:  *P_k_* = *U*Σ̂*V^T^* *Bp_k_* ← *Bp_k_*_−1_ + *X_k_* − *P_k_*

This algorithm solves the unconstrained problem (24). The final task is to solve the constrained one (20). This is achieved by cooling. The algorithm is given below. The algorithm starts with a high value of *λ*. In the outer loop, the value of *λ* is decreased (cooled), while in the inner loop the unconstrained problem is solved.

Start with a high value of *λ*Continue outer loop till *λ* > *Tol*Inner loop iteration (k):
$X_{k}\leftarrow \underset{X}{\min}{\left\|Y-M(X)\right\|}_{\text{Fro}}^{2}+\gamma {\left\|P-X-Bp\right\|}_{\text{Fro}}^{2}$
$P_{k}\leftarrow \underset{P}{\min}\lambda {\left\|P\right\|}_{S_{p}}^{p}+\gamma {\left\|P-X-Bp\right\|}_{\text{Fro}}^{2}$  Compute SVD of *X* + *Bp*, *i.e.*, *U*Σ*V^T^* = *X_k_*_−1_ + *Bp_k_*_−1_.  Soft threshold the singular values: 
$\hat{\sum }=\text{sigum}(\text{diag}(\sum ))(0,\text{diag}(\sum )-\frac{\lambda }{2\alpha }\text{diag}(\sum ).^{\left(p-2\right)})$ Low-rank approximation:  *P_k_* = *U*Σ̂*V^T^**Bp_k_* ← *Bp_k_*_−1_ + *X_k_* − *P_k_*Inner loop endsFor outer loop: *λ* ← *λ*×*DecFac*

Our algorithm requires specifying the value of γ. We fix it at 10^−3^. The relaxation variable Bp is initialized to all ones and P is initialized to all zeros. There are two exit criteria for the inner loop. Either it continues till convergence, *i.e.*, the objective function between two successive iterations is smaller than a certain tolerance (10^−4^ in our case), or it continues for a maximum number of iterations (50). The outer loop exits until the error reached is such that ${\left\|B-R⊙X\right\|}_{\text{Fro}}^{2}\geq \varepsilon$.

## 6. Experimental Section

### 6.1. Experiments on Synthetic Data

For testing our proposed algorithm—Matrix Completion via the Split Bregman technique (MSB), matrices of dimensions 250 × 250 and of varying ranks were constructed. The matrices were formed by multiplying two i.i.d Gaussian matrices of same rank. They were sub-sampled at varying sampling ratios. The value of p (for Schatten-p norm) was fixed at 0.6. However, we found that the results were robust to variations for a value of p between 0.3 and 0.8; the results degrade gracefully outside this range.

We compared our results against those obtained using Singular Value Thresholding (SVT) [23] and Fixed Point Continuation (FPC) [24]. For FPC, the value of mu_final was taken to be 0.01 and the tolerance was 10^−3^. For SVT, the maximum number of iterations was taken to be 500. These values yielded the best results for the corresponding algorithms.

**Figure 3.**
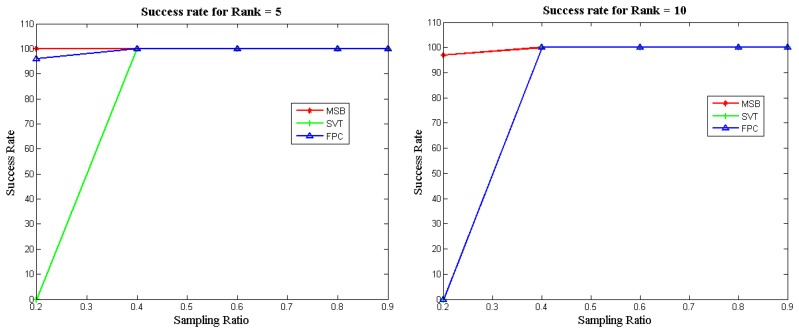

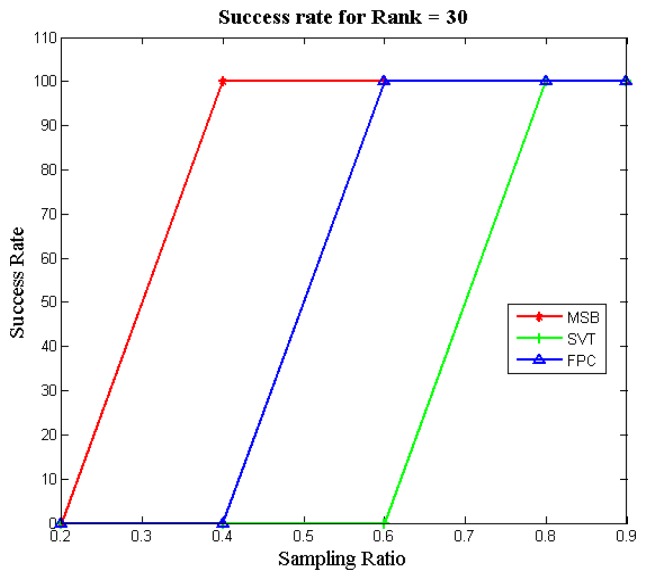
Empirical Success Rates.

Figure 3 illustrates the success rates of the FPC, SVT and MSB algorithms as a function of the sampling ratio for different matrix ranks. To compute the success rate, 100 datasets were generated for each configuration (*i.e.*, sampling ratio and rank) and independent runs of each algorithm were carried out. The success rate was computed as the number of attempts (out of 100) which achieved a Normalized Mean Squared Error (NMSE) less than 10^−3^. It can be seen that our proposed approach (MSB) always yields the best recovery; *i.e.*, it can successfully recover the matrices obtained by low sampling ratios, unlike the other two methods.

We also carried out experiments with the IRPF (Incremental Rank Power Factorization) [25] and IRLS (Iterative Reweighted Least Squares) [26] techniques. However, these techniques could not get the error to be less than 10^−3^ for any sampling ratio. Therefore we did not report the results in Figure 3. However, we report the results from these algorithms in the following tables.

The run times for all three algorithms are shown in Table 1. The table clearly shows that our algorithm converges faster to the optimum value. The execution time for MSB is much lower than all the algorithms compared against, especially for matrices with high ranks and very few (available) sampled values.

The success rate gives an overall picture. However, to assess the recovery accuracy, the use of the success rate graphs alone is not enough. We need to observe the reconstruction errors. For this purpose, the average (Normalized Mean Squared Error) NMSE for all three algorithms (MSB, SVT, IRPF, IRLS and FPC), computed over 100 independent trials for each configuration (rank and sampling ratio), is shown in Table 2. It is evident from the numerical values that our algorithm provides much lower NMSE, in all the cases; it is at least an order of magnitude smaller for most cases.

### 6.2. Experiments on EEG Signals

There is no actual benchmark to compare our proposed method. Compressed Sensing based methods are incapable of operating in the sensing paradigm where the EEG signal is under-sampled in the time domain. However in order to test our method, we compare it to two state-of-the-art CS based recovery schemes—sparse recovery [8] and BSBL [9]. Both these methods use random binary projection matrices for compressing the EEG signal. It should be kept in mind, that these CS methods do not save sensing power. They only save transmission power.

We also carried out experiments to recover the under-sampled EEG signals by sparse recovery and BSBL. However, the results are considerably poorer. This is expected following our discussion; such CS based techniques are suitable for recovering the signal from random under-sampling. To give a typical example, for 40% under-sampling/compression, the error increases from 0.11 to 0.18 for BSBL and from 0.14 to 0.22 for sparse recovery when the measurement basis changes from sparse binary random matrices to random under-sampling. As the results are significantly poorer, we do not show them in the paper.

The experiments are carried out on two datasets—the BCI Competition III dataset [27] and the EEGLab dataset [28]. The BCI competition dataset was actually used for binary classification problems. For more details, please peruse the aforesaid reference. We tested the recovery results for various sampling ratios. We found that the best results were obtained for *p* = *0.6*. The Normalized Mean Squared Error (NMSE) is used as the standard metric for reconstruction accuracy for such problems.

**Figure 4.**
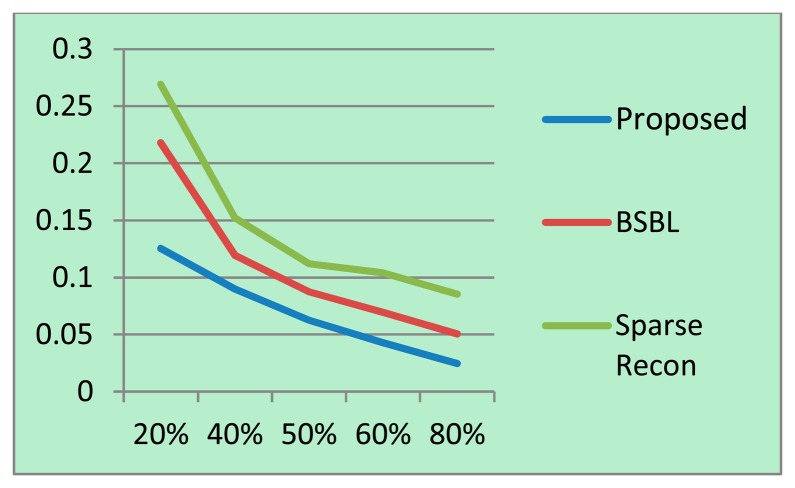
BCI dataset: NMSE on *Y*-axis, sampling/compression ratio on *X*-axis.

For each sampling/compression ratio, the experiments were run 100 times and the mean NMSE is plotted for different sampling/compression ratios in Figures 4 (BCI) and 5 (EEGLab). It should be kept in mind that “sampling ratio” only pertains to our proposed method; the other methods sample the full signal and compress it later and hence is plotted against “compression ratio”.

**Figure 5.**
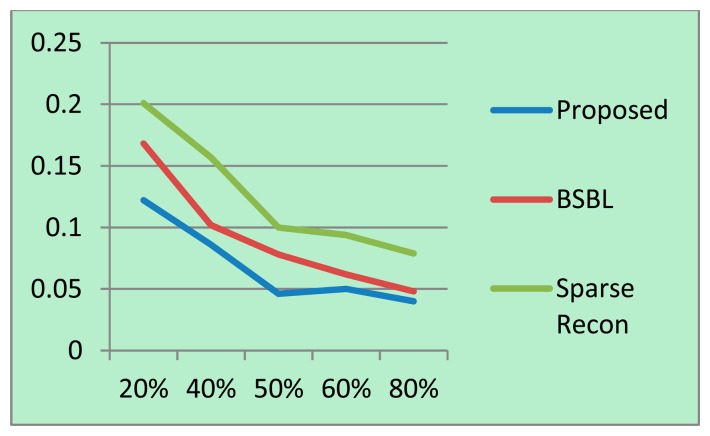
EEGLab dataset: NMSE on *Y*-axis, sampling/compression ratio on *X*-axis.

**Figure 6.**
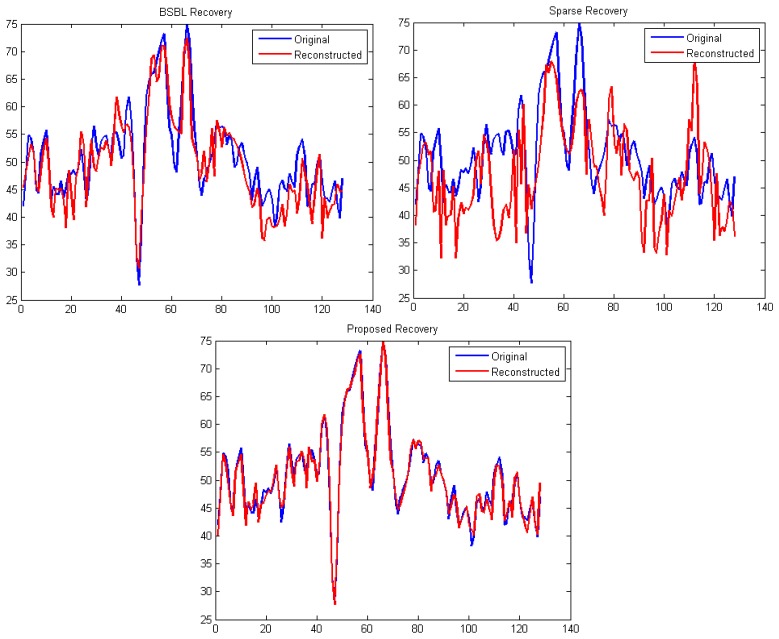
Overlayed original and reconstructed signals from different techniques (BSBL, sparse reconstruction and proposed). Sampling instants (time points) on the *X*-axis and signal amplitude on the *Y*-axis.

It can be easily seen from Figures 4 and 5 that our proposed method consistently yields the best reconstruction for all sampling ratios. For qualitative evaluation, we show an original and the corresponding reconstructed signals in Figure 6. These signals are from the BCI dataset.

It can be seen that BSBL reconstruction [9] and sparse reconstruction [8] show certain artifacts. Such artifacts are not visible in our proposed reconstruction. Thus, our proposed method not only reduces sensing energy but also yields better reconstruction compared to prior CS based techniques (which do not reduce sensing energy).

NMSE is one of the best known measures for accuracy, especially for CS reconstruction. However, reconstruction is not the end of the information processing pipeline. Some automated or manual analysis ensues. In this paper, we wanted to check how the different reconstruction methods perform on a standard automated analysis task such as classification. We carried out the classification using algorithm [29] on the BCI III dataset. The classification was carried out on the groundtruth as well as on the reconstructed EEG data. The classification results are shown in the following table (Table 3).

The classification algorithm when applied on the groundtruth dataset yields a reconstruction accuracy of 81% (the same result is reported in the BCI III competition website [29]). When the same algorithm is applied on the reconstructed data, our method yields the best accuracy; the classification accuracy is slightly lower compared to the ground-truth. The classification accuracy from the other methods [8,9] is slightly lower compared to ours.

## 7. Conclusions

Compressed Sensing based techniques, as developed so far, for energy efficient transmission of EEG signals on WBANs, can only reduce the transmission energy costs. This paper proposes, for the first time, the reduction in sensing energy. This automatically results in a reduction in the processing and also transmission energy costs. As CS based methods cannot be used to reduce the sensing energy, we proposed an alternate formulation based on low-rank matrix recovery to reconstruct all the EEG signals from all the channels simultaneously.

The results show that our proposed method gives better reconstruction results than state-of-the-art CS based techniques [8,9]. The biggest advantage of our method is not only the improvement in accuracy, but the fact that we are able to get more accurate results with lower expenditure of sensing power.

We carried out a thorough power analysis to verify the power saving enabled by our proposed scheme. We find that our proposed scheme will consume considerably less power compared to previous CS based techniques.

However, the proposed method has a shortcoming. The assumption that the EEG signal matrix is low-rank only applies when the number of channels are large; in situations where the number of channels are small, the low-rank assumption will not hold. We experimentally found that the number of channels should be at least 16 for this method to work. However, this is not a serious shortcoming since most modern EEG sensing devices have more channels than required by our method.

## Figures and Tables

**Table 1.** Run times for different algorithms.

**Rank**	**Algorithm**	**Sampling Ratios**	

		**0.2**	**0.4**
40	FPC	46.4	338.7
	SVT	447.2	372.6
	IRPF	328.3	300.9
	IRLS	559.7	507.2
	MSB	**32.8**	**34.1**

30	FPC	38.8	189.7
	SVT	406	179.3
	IRPF	300.1	256.6
	IRLS	502.3	447.3
	MSB	**26.5**	**44.2**

20	FPC	34.12	21.9
	SVT	342	31.7
	IRPF	259.8	198.9
	IRLS	400.9	337.3
	MSB	**20.7**	**63.3**

**Table 2.** NMSE for different algorithms.

**Rank**	**Algorithm**	**Sampling Ratios**			

		**0.2**	**0.4**	**0.6**	**0.8**
5	FPC	5.1E−4	2.2E−4	1E−4	8.5E−5
	SVT	1.6E−3	2.1E−4	1.1E−4	1.01E−4
	IRPF	1.1E−1	5.2E−2	1.8E−2	6.9E−3
	IRLS	2.4E−2	1.0E−2	5.7E−3	1.9E−3
	MSB	**6.1E−6**	**4.15E−7**	**1.06E−7**	**5.27E−8**

10	FPC	1.67E−2	2E−4	8.99E−5	6.31E−5
	SVT	8.8E−3	6E−4	1E−4	1E−4
	IRPF	2.0E−1	6.1E−2	2.8E−2	5.0E−3
	IRLS	6.4E−2	5.1E−2	1.7E−2	7.2E−2
	MSB	**2.45E−4**	**4.58E−7**	**5.89E−8**	**5.43E−8**

20	FPC	4.19E−2	2.1E−4	7.31E−5	4.74E−5
	SVT	7.19E−2	2.3E−3	4.3E−4	1.3E−4
	IRPF	2.8E−1	7.82E−2	4.88E−2	7.0E−3
	IRLS	8.0E−2	6.09E−2	2.73E−2	4.2E−3
	MSB	**1.81E−2**	**7.09E−6**	**6.96E−6**	**1.28E−6**

30	FPC	4.39E−2	4.3E−3	6.98E−5	4.11E−5
	SVT	8.06E−2	1.18E−2	1.1E−3	2.1E−4
	IRPF	4.8E−1	1.8E−1	9.87E−2	1.0E−2
	IRLS	1.31E−1	8.9E−2	6.7E−2	8.2E−3
	MSB	**4.33E−2**	**7.42E−4**	**9.43E−6**	**1.80E−6**

40	FPC	4.4E−2	1.5E−2	7.44E−5	3.04E−5
	SVT	8.5E−2	2.78E−2	2.1E−3	1.6E−4
	IRPF	9.0E−1	3.8E−1	1.7E−1	6.0E−2
	IRLS	5.6E−1	1.9E−1	9.5E−2	1.2E−2
	MSB	**4.1E−2**	**4.4E−3**	**3.58E−5**	**1.78E−5**

**Table 3.** Classification accuracy in %.

**Method**	**Classification Accuracy**	

	**Sampling/Compression 2:1**	**Sampling/Compression 4:1**
Groundtruth	81% (No Compression)	

BSBL	76%	54%
Sparse Recon	74%	50%
Proposed	**78%**	**57%**
